# Enteric motor pattern generators involve both myogenic and neurogenic mechanisms in the human colon

**DOI:** 10.3389/fphys.2015.00205

**Published:** 2015-07-21

**Authors:** Noemí Mañé, Míriam Martínez-Cutillas, Diana Gallego, Marcel Jimenez

**Affiliations:** ^1^Cell Biology, Physiology and Immunology, Universidad Autonoma de BarcelonaBarcelona, Spain; ^2^Centro de Investigación Biomédica en Red de Enfermedades Hepáticas y DigestivasBarcelona, Spain

**Keywords:** human colonic motility, Interstitial Cells of Cajal, enteric neurotransmission, *in vitro* motility patterns, pacemaker

Coordinated motor activity is required to develop the major functions of the colon, which are: 1-absorption of water, electrolytes, bile salts, short-chain fatty acids and other bacterial metabolites, 2-storage of colonic contents and 3-propulsion of fecal material (Christensen, 1991). Interstitial cells of Cajal (ICCs) generate spontaneous pacemaker currents which are conducted to smooth muscle cells (SMCs) causing rhythmic contractile patterns (Rumessen et al., 1993; Huizinga et al., 1995). Even though *in vitro* experiments disrupt enteric neural pathways crucial to develop a variety of *in vivo* colonic motor patterns and rule out any influence of extrinsic innervation, they are useful to better understand the mechanisms underlying colonic motility. Accordingly, the aim of this article is to summarize myogenic and neurogenic activities described in the human colon, hypothesize about how these mechanisms might be related and propose a new concept, *enteric motor pattern generators*, for this interplay.

Circularly-oriented strips from the human colon most commonly display low-amplitude contractions at an equivalent frequency to slow waves (Figure 1A). Strips that preserve the submucous plexus exhibit prominent 2–4 c.p.m slow waves that have their greatest amplitude near the ICC-submuscular plexus (ICC-SMP) (Rae et al., 1998). ICC-SMP are therefore responsible for colonic slow waves in the colon of animals (Langton et al., 1989; Pluja et al., 2001) and humans (Rumessen et al., 1993). Septal ICCs described in the human colon (Liu et al., 2012) might spread this pacemaker activity as previously suggested in the human small intestine (Lee et al., 2007a,b). *In vivo*, rhythmic phasic contractions (RPCs) at a frequency of 2–4 c.p.m are commonly recorded in the human large intestine (Taylor et al., 1975; Latimer et al., 1981; Narducci et al., 1987; Ford et al., 1995) strongly correlating to their frequency *in vitro*. The classical view states that the main role of RPCs is the turning over and mixing of luminal contents. However, recent studies using high resolution manometry have shown that RPCs can propagate anally or orally over short distances possibly causing propulsion (Dinning et al., 2014).

**Figure 1:**
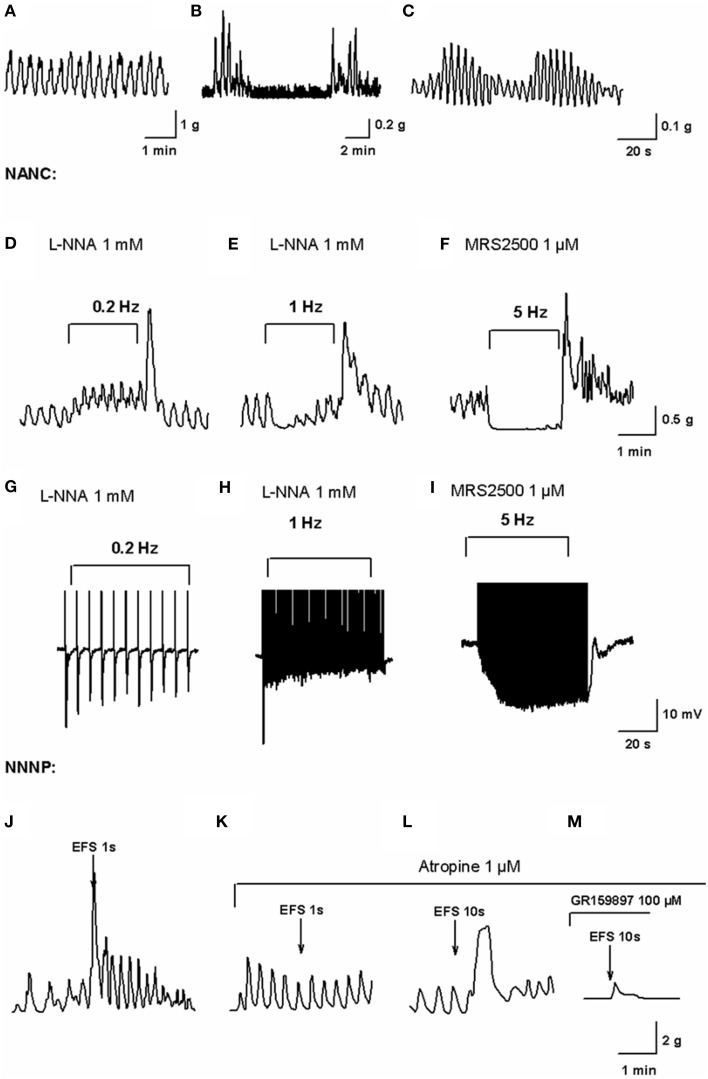
***In vitro*** **motility patterns and enteric neurotransmission in the human colon**. Mechanical recording of high frequency (HF) contractions of a constant amplitude probably associated to slow wave activity **(A)**, low-frequency (LF) contractions superimposed to HF contractions **(B)** and wax and wane of HF contractions amplitude **(C)** obtained from the experiments performed in our lab (Mane et al., 2014a). Mechanical recording of EFS under NANC conditions on L-NNA 1 mM incubated tissue at a frequency of 0.2 Hz **(D)** and 1 Hz **(E)** and MRS2500 1 μM incubated tissue at a frequency of 5 Hz **(F)** (Mane et al., 2014a). In L-NNA incubated tissue, purinergic neurotransmission is only able to cause phasic relaxations while nitrergic neurotransmission at 5 Hz completely inhibits spontaneous contractions. Electrophysiological recording of electrical field stimulation on L-NNA incubated tissue at a frequency of 0.2 Hz **(G)** and 1 Hz **(H)** and MRS2500 incubated tissue at a frequency of 5 Hz **(I)** (Mane et al., 2014a). Notice how purinergic fast IJP amplitude is reduced with high frequencies of EFS after the first pulse while the nitrergic response increases due to summation of slow IJP. Mechanical recording of EFS in NNNP conditions at 50 Hz, 50 V, 0.4 ms for 1 s **(J)** eliciting an atropine-sensitive **(K)** contraction. Mechanical recording of EFS in NNNP conditions at 50 Hz, 50 V, 0.4 ms for 10 s **(L)** eliciting an antiNK2-sensitive **(M)** contraction (Martinez-Cutillas et al., 2015).

RPCs can appear together with high-amplitude contractions of a lower frequency (Figure 1B). When Rae et al. (1998) removed the submucosal plexus from human colonic strips leaving the myenteric plexus intact, strips displayed tetrodotoxin (TTX)-insensitive high-amplitude contractions at a frequency of 0.3–0.6 c.p.m. A similar contractile pattern has been reported in rat and mouse colonic strips when the ICC-SMP is removed (Pluja et al., 2001; Domenech et al., 2011). These results demonstrate the presence of a second myogenic pacemaker not dependent on the ICC-SMP or the enteric nervous system. The most probable origin of this pacemaker is the ICC network located near the myenteric plexus (ICC-MP) (Pluja et al., 2001; Carbone et al., 2013). In colonic large segments of different species, propulsive contractions at an identical frequency can be recorded. These propulsive contractions are inhibited after neural blockade but they can be restored with subsequent addition of carbachol demonstrating that myogenic propulsive contractions can occur in species such as the rat and rabbit colon (Huizinga et al., 2011; Costa et al., 2013). The ICC-MP receives excitatory neural inputs (Faussone-Pellegrini et al., 1990; Bayguinov et al., 2010) that may trigger the appearance of the low-frequency pacemaker *in vivo*. In *in vitro* conditions, the mechanisms responsible for the development of low-frequency contractions might be the mechanical stimuli elicited by the stretching of the strip (Huizinga et al., 2011).

Propagating spontaneous colonic motor complexes (CMCs) are the principal motor pattern occurring in *in vitro* recordings of whole isolated human colon (Spencer et al., 2012). They occur approximately every 4 min (0.25 c.p.m.), a frequency similar to the one of the low-frequency contractions mentioned above. Possibly due to the dimensions of the organ bath and the derived technical difficulties, no experimental evidences were provided about the neural origin of this activity, which has been confirmed in the mouse colon (Bywater et al., 1989).

High resolution manometry revealed mainly four contractile colonic motor patterns in humans (Dinning et al., 2014): cyclic motor patterns corresponding to RPCs (slow waves), short and long single propagating motor patterns and high-amplitude propagating contractions (HAPCs). Short and long single propagating contractions appear in intervals of more than 1 min, a frequency similar to the one displayed by low-frequency contractions recorded *in vitro*. HAPCs appear post-pandrially and only represent a small percentage of the total motor patterns recorded in healthy humans (6–10 HAPCs in 24 h, Bassotti and Gaburri, 1988; Rao et al., 2001; De Schryver et al., 2002). These high amplitude contractions develop a propulsive role (Cook et al., 2000), propelling intraluminal contents over large distances (Cannon, 1902). HAPCs can be activated by mechanical stimulation or chemical stimuli acting on underlying neural circuits which then initiate self-sustaining HAPCs. These activation mechanisms are similar to the ones that trigger low-frequency contractions in rat colonic segments. Once initiated, HAPCs cannot be blocked by lignocaine (Hardcastle and Mann, 1968) and therefore do not need a neural circuitry to develop after activation. We believe that the low-frequency pacemaker is the electrophysiological basis of short and long single propagating contractions and HAPCs. Luminal and extraluminal inputs may activate enteric neurons that in turn enhance the pacemaker possibly displayed by the ICC-MP. Consequently the force of contractions can increase from ≈23 mmHg of single propagating contractions to the ≈240 mmHg of HAPCs. Alternatively, inhibitory neurons can inhibit this myogenic pacemaker leaving RPCs as the predominant pattern.

As previously described in the rat colon (Mane and Jimenez, 2014), human colonic strips can display a “wax and wane” pattern of RPCs (Figure 1C) that can also be observed in the small intestine (see Figure 4 in Gallego et al., 2014). This motor pattern has been stated to be the basis of a segmentation-like pattern in the murine small intestine and the rat colon (Huizinga et al., 2014; Mane and Jimenez, 2014) and modulation of slow wave amplitude by a second pacemaker of a lower frequency has been proposed to be the underlying mechanism responsible. This hypothesis was raised due to the fact that both the ICC-deep muscular plexus of the murine small intestine and the ICC-MP of the rat colon display a pacemaker of an identical frequency to the wax and wane. Curiously enough, in the human colon the frequency of the wax and wane is similar to the frequency of low-frequency contractions. We therefore propose that in the human colon, the low-frequency pacemaker is the basis of the cyclic decrease in the amplitude of slow wave activity. Alternate contraction and relaxation of colonic segments separate the colon into chambers facilitating the contact between the intraluminal content and the colonic wall favoring absorption, mixing and slow propulsion of colonic contents.

The storage of colonic contents for long periods is accomplished by a sustained inhibition of contractile activity probably related to the presence of a neural inhibitory tone. Both in the human colon (Rae et al., 1998; Gallego et al., 2008) and laboratory animals (Alberti et al., 2005; Gallego et al., 2012), the addition of TTX usually increases the amplitude and frequency of spontaneous contractions *in vitro*. A similar effect is observed when colonic tissue is incubated with L-NNA or ODQ, a nitric oxide (NO) synthase and a guanylyl cyclase (Gc) inhibitor respectively. Therefore, a constant *in vitro* inhibition of spontaneous motility as a consequence of the “spontaneous” release of NO from enteric neurons has been reported. We believe that by stretching colonic strips in both electrophysiological and mechanical experiments, the release of NO is increased as the stretching may mimic the distention elicited by luminal content.

Excitatory and inhibitory neurons have, therefore, a role in the generation and/or development of the motor patterns explained above. While inhibitory neurotransmission is responsible for sustained relaxations of the colon, an active participation of excitatory neurotransmission has been proposed in the triggering of low-frequency contractions, development of CMC and HAPCs. Enteric innervation of the ICC networks can modulate the predominant motility pattern displayed by a certain colonic area in order to favor the development of the desired colonic function. Moreover, excitatory and inhibitory neurons can relax or contract the tissue independently of pacemaker activity.

*In vitro*, electrical field stimulation (EFS) of human colonic tissue elicits complex responses including contractions, relaxations and consequent off-contractions. These responses are the result of the simultaneous stimulation of all enteric neuronal pathways. As this might never happen *in vivo*, pharmacological conditions and parameters for selective stimulation of concrete neural pathways have to be established in order to emulate *in vivo* neurotransmission as accurately as possible.

Until now, in the human colon, enteric inhibitory neurotransmission has been shown to involve mainly purines and NO. It is well known that non-adrenergic, non-cholinergic (NANC) conditions are needed to study inhibitory neurotransmission. To further isolate purinergic and nitrergic responses, the NO synthesis blocker L-NNA or the P2Y_1_ antagonist MRS2500 should be used. The frequency of EFS, which we believe mimics the firing frequency of neurons, has been stated to be crucial to enhance one or another component of inhibitory neurotransmission: while purinergic responses are dominant at low frequencies (<1 Hz) (Figure 1G) or short bursts (Figure 1H), high frequencies (>1 Hz) of EFS are needed to release NO (Figure 1I) (Mane et al., 2014a,b). Purinergic responses are attenuated in a frequency-dependent manner and are therefore responsible for phasic relaxations required in propulsive activity (Figures 1D,E). NO, in contrast, can cause long-lasting inhibition of myogenic contractions (Figure 1F) and is therefore crucial for storage functions (see above).

On the other hand, the major neurotransmitters responsible for contractions released by enteric excitatory neurons are acetylcholine and tachykinins. Excitatory neurotransmission should be always characterized under non-nitrergic (L-NNA), non-purinergic (MRS2500) (NNNP) conditions and in presence of propranolol and phentolamine. In this case, the duration of the neuronal burst determines if the response is mainly cholinergic (short burst, 1 s) (Figures 1J,K) or if it also involves the release of tachykinins (long bursts, 10 s) (Figures 1L,M) (Martinez-Cutillas et al., 2015).

Direct innervation of SMCs by enteric motoneurons has been discussed over the last years. Interstitial cells have been proposed to mediate neurotransmission in the gut due to their proximity to nerve endings, their expression of receptors and signaling pathways for neurotransmitters and the existence of gap junctions with SMCs. Concerning excitatory neurotransmission, it has been shown that the ICC-MP receives excitatory input from motoneurons that release acetylcholine and tachykinins (Bayguinov et al., 2010) and that these cells are required for the mediation of cholinergic post-junctional responses. In nitrergic neurotransmission, guanylyl cyclase in both SMCs and ICC has been proven to be mandatory to induce a full nitrergic IJP (Lies et al., 2014). More recently, platelet derived growth factor receptor α positive (PDGFRα+) cells in colonic muscles (Kurahashi et al., 2012), which are also innervated by enteric inhibitory motoneurons, have been shown to mediate purinergic neurotransmission (Kurahashi et al., 2014). All these data have been provided using genetic modified mice that lack ICC or a certain post-junctional pathway, but the role of interstitial cells including PDGFRα+ cells in human tissue needs further evaluation.

Until now, the most studied intestinal motility pattern has been the peristaltic reflex. However, although a polarization of intrinsic neural pathways has been shown to exist in the human colon (Porter et al., 2002), very poor responses to acute distension have been reported using whole colonic preparations (Spencer et al., 2012). Therefore, it is unreasonable to believe that the peristaltic reflex is the unique basis of colonic motility. This would be like stating that spinal reflexes are the basis of movement. We believe that like spinal reflexes, peristaltic reflexes are only activated by particular stimuli. We propose a new concept, “enteric motor pattern generator” as an equivalent to the “central pattern generators” described in the spinal cord. In the gastrointestinal tract, different sublclasses of interstitial cells are the source of primary motility pacing contractions occurring at different frequencies. These patterns are constantly modulated by intrinsic and extrinsic innervation that provide the essential neural input to develop an efficient gastrointestinal motility.

## Conflict of interest statement

The authors declare that the research was conducted in the absence of any commercial or financial relationships that could be construed as a potential conflict of interest.

## References

[B1] Alberti E. Mikkelsen H. B. Larsen J. O. Jimenez M. (2005). Motility patterns and distribution of interstitial cells of Cajal and nitrergic neurons in the proximal, mid- and distal-colon of the rat. Neurogastroenterol. Motil. 17, 133–147. 10.1111/j.1365-2982.2004.00603.x 15670273

[B2] Bassotti G. Gaburri M. (1988). Manometric investigation of high-amplitude propagated contractile activity of the human colon. Am. J. Physiol. 255, G660–G664. 3189553 10.1152/ajpgi.1988.255.5.G660

[B3] Bayguinov P. O. Hennig G. W. Smith T. K. (2010). Ca2+ imaging of activity in ICC-MY during local mucosal reflexes and the colonic migrating motor complex in the murine large intestine. J. Physiol. 588, 4453–4474. 10.1113/jphysiol.2010.196824 20876203 PMC3008851

[B4] Bywater R. A. Small R. C. Taylor G. S. (1989). Neurogenic slow depolarizations and rapid oscillations in the membrane potential of circular muscle of mouse colon. J. Physiol. 413, 505–519. 10.1113/jphysiol.1989.sp017666 2600862 PMC1189113

[B5] Cannon W. B. (1902). The Movements of the Intestines studied by Means of the Rontgen Rays. J. Med. Res. 7, 72–75. 19971454 PMC2105834

[B6] Carbone S. E. Dinning P. G. Costa M. Spencer N. J. Brookes S. J. Wattchow D. A. (2013). Ascending excitatory neural pathways modulate slow phasic myogenic contractions in the isolated human colon. Neurogastroenterol. Motil. 25, 670–676. 10.1111/nmo.12129 23634776

[B7] Christensen J. (1991). Gross and microscopic anatomy of the large intestine, in The Large Intestine - Physiology, Pathophysiology and Disease, eds Phillips S. F. Pemberton J. H. Shorter R. G. (New York, NY: Reven press), 13–35.

[B8] Cook I. J. Furukawa Y. Panagopoulos V. Collins P. J. Dent J. (2000). Relationships between spatial patterns of colonic pressure and individual movements of content. Am. J. Physiol. Gastrointest. Liver Physiol. 278, G329–G341. 10666058 10.1152/ajpgi.2000.278.2.G329

[B9] Costa M. Dodds K. N. Wiklendt L. Spencer N. J. Brookes S. J. Dinning P. G. (2013). Neurogenic and myogenic motor activity in the colon of the guinea pig, mouse, rabbit, and rat. Am. J. Physiol. Gastrointest. Liver Physiol. 305, G749–G759. 10.1152/ajpgi.00227.2013 24052530

[B10] De Schryver A. M. Samsom M. Smout A. J. (2002). In search of objective manometric criteria for colonic high-amplitude propagated pressure waves. Neurogastroenterol. Motil. 14, 375–381. 10.1046/j.1365-2982.2002.00342.x 12213105

[B11] Dinning P. G. Wiklendt L. Maslen L. Gibbins I. Patton V. Arkwright J. W.. (2014). Quantification of *in vivo* colonic motor patterns in healthy humans before and after a meal revealed by high-resolution fiber-optic manometry. Neurogastroenterol. Motil. 26, 1443–1457. 10.1111/nmo.12408 25131177 PMC4438670

[B12] Domenech A. Pasquinelli G. De G. R. Gori A. Bosch F. Pumarola M.. (2011). Morphofunctional changes underlying intestinal dysmotility in diabetic RIP-I/hIFNbeta transgenic mice. Int. J. Exp. Pathol. 92, 400–412. 10.1111/j.1365-2613.2011.00789.x 22050417 PMC3248076

[B13] Faussone-Pellegrini M. S. Pantalone D. Cortesini C. (1990). Smooth muscle cells, interstitial cells of Cajal and myenteric plexus interrelationships in the human colon. Acta Anat. (Basel.) 139, 31–44. 10.1159/000146975 2288187

[B14] Ford M. J. Camilleri M. Wiste J. A. Hanson R. B. (1995). Differences in colonic tone and phasic response to a meal in the transverse and sigmoid human colon. Gut 37, 264–269. 10.1136/gut.37.2.264 7557579 PMC1382729

[B15] Gallego D. Gil V. Aleu J. Auli M. Clave P. Jimenez M. (2008). Purinergic and nitrergic junction potential in the human colon. Am. J. Physiol. Gastrointest. Liver Physiol. 295, G522–G533. 10.1152/ajpgi.00510.2007 18599588

[B16] Gallego D. Gil V. Martinez-Cutillas M. Mane N. Martin M. T. Jimenez M. (2012). Purinergic neuromuscular transmission is absent in the colon of P2Y(1) knocked out mice. J. Physiol. 590, 1943–1956. 10.1113/jphysiol.2011.224345 22371472 PMC3573314

[B17] Gallego D. Malagelada C. Accarino A. De G. R. Malagelada J. R. Azpiroz F.. (2014). Nitrergic and purinergic mechanisms evoke inhibitory neuromuscular transmission in the human small intestine. Neurogastroenterol. Motil. 26, 419–429. 10.1111/nmo.12293 24372768

[B18] Hardcastle J. D. Mann C. V. (1968). Study of large bowel peristalsis. Gut 9, 512–520. 10.1136/gut.9.5.512 5717099 PMC1552760

[B19] Huizinga J. D. Chen J. H. Zhu Y. F. Pawelka A. McGinn R. J. Bardakjian B. L.. (2014). The origin of segmentation motor activity in the intestine. Nat. Commun. 5, 3326. 10.1038/ncomms4326 24561718 PMC4885742

[B20] Huizinga J. D. Martz S. Gil V. Wang X. Y. Jimenez M. Parsons S. (2011). Two independent networks of interstitial cells of cajal work cooperatively with the enteric nervous system to create colonic motor patterns. Front. Neurosci. 5:93. 10.3389/fnins.2011.00093 21833164 PMC3153851

[B21] Huizinga J. D. Thuneberg L. Kluppel M. Malysz J. Mikkelsen H. B. Bernstein A. (1995). W/kit gene required for interstitial cells of Cajal and for intestinal pacemaker activity. Nature 373, 347–349. 10.1038/373347a0 7530333

[B22] Kurahashi M. Mutafova-Yambolieva V. Koh S. D. Sanders K. M. (2014). Platelet-derived growth factor receptor-alpha-positive cells and not smooth muscle cells mediate purinergic hyperpolarization in murine colonic muscles. Am. J. Physiol. Cell Physiol. 307, C561–C570. 10.1152/ajpcell.00080.2014 25055825 PMC4166738

[B23] Kurahashi M. Nakano Y. Hennig G. W. Ward S. M. Sanders K. M. (2012). Platelet-derived growth factor receptor alpha-positive cells in the tunica muscularis of human colon. J. Cell Mol. Med. 16, 1397–1404. 10.1111/j.1582-4934.2011.01510.x 22225616 PMC3477549

[B24] Langton P. Ward S. M. Carl A. Norell M. A. Sanders K. M. (1989). Spontaneous electrical activity of interstitial cells of Cajal isolated from canine proximal colon. Proc. Natl. Acad. Sci. U.S.A. 86, 7280–7284. 10.1073/pnas.86.18.7280 2550938 PMC298041

[B25] Latimer P. Sarna S. Campbell D. Latimer M. Waterfall W. Daniel E. E. (1981). Colonic motor and myoelectrical activity: a comparative study of normal subjects, psychoneurotic patients, and patients with irritable bowel syndrome. Gastroenterology 80, 893–901. 7202974

[B26] Lee H. T. Hennig G. W. Fleming N. W. Keef K. D. Spencer N. J. Ward S. M.. (2007a). Septal interstitial cells of Cajal conduct pacemaker activity to excite muscle bundles in human jejunum. Gastroenterology 133, 907–917. 10.1053/j.gastro.2007.06.024 17678922 PMC2077833

[B27] Lee H. T. Hennig G. W. Fleming N. W. Keef K. D. Spencer N. J. Ward S. M.. (2007b). The mechanism and spread of pacemaker activity through myenteric interstitial cells of Cajal in human small intestine. Gastroenterology 132, 1852–1865. 10.1053/j.gastro.2007.02.049 17484879

[B28] Lies B. Gil V. Groneberg D. Seidler B. Saur D. Wischmeyer E.. (2014). Interstitial cells of Cajal mediate nitrergic inhibitory neurotransmission in the murine gastrointestinal tract. Am. J. Physiol. Gastrointest. Liver Physiol. 307, G98–G106. 10.1152/ajpgi.00082.2014 24833707

[B29] Liu Y. A. Chung Y. C. Pan S. T. Hou Y. C. Peng S. J. Pasricha P. J.. (2012). 3-D illustration of network orientations of interstitial cells of Cajal subgroups in human colon as revealed by deep-tissue imaging with optical clearing. Am. J. Physiol. Gastrointest. Liver Physiol. 302, G1099–G1110. 10.1152/ajpgi.00432.2011 22421617 PMC3362097

[B30] Mane N. Gil V. Martinez-Cutillas M. Clave P. Gallego D. Jimenez M. (2014a). Differential functional role of purinergic and nitrergic inhibitory cotransmitters in human colonic relaxation. Acta Physiol. (Oxf.) 212, 293–305. 10.1111/apha.12408 25327170

[B31] Mane N. Gil V. Martinez-Cutillas M. Martin M. T. Gallego D. Jimenez M. (2014b). Dynamics of inhibitory co-transmission, membrane potential and pacemaker activity determine neuromyogenic function in the rat colon. Pflugers Arch. 466, 2305–2321. 10.1007/s00424-014-1500-8 24658973

[B32] Mane N. Jimenez M. (2014). Interplay between myogenic pacemakers and enteric neurons determine distinct motor patterns in the rat colon. Neurogastroenterol. Motil. 26, 1508–1512. 10.1111/nmo.12393 25088991

[B33] Martinez-Cutillas M. Gil V. Mane N. Clave P. Gallego D. Martin M. T.. (2015). Potential role of the gaseous mediator hydrogen sulphide (HS) in inhibition of human colonic contractility. Pharmacol. Res. 93C, 52–63. 10.1016/j.phrs.2015.01.002 25641403

[B34] Narducci F. Bassotti G. Gaburri M. Morelli A. (1987). Twenty four hour manometric recording of colonic motor activity in healthy man. Gut 28, 17–25. 10.1136/gut.28.1.17 3817580 PMC1432711

[B35] Pluja L. Alberti E. Fernandez E. Mikkelsen H. B. Thuneberg L. Jimenez M. (2001). Evidence supporting presence of two pacemakers in rat colon. Am. J. Physiol. Gastrointest. Liver Physiol. 281, G255–G266. 11408279 10.1152/ajpgi.2001.281.1.G255

[B36] Porter A. J. Wattchow D. A. Brookes S. J. Costa M. (2002). Cholinergic and nitrergic interneurones in the myenteric plexus of the human colon. Gut 51, 70–75. 10.1136/gut.51.1.70 12077095 PMC1773285

[B37] Rae M. G. Fleming N. McGregor D. B. Sanders K. M. Keef K. D. (1998). Control of motility patterns in the human colonic circular muscle layer by pacemaker activity. J. Physiol. 510(Pt 1), 309–320. 10.1111/j.1469-7793.1998.309bz.x 9625887 PMC2231034

[B38] Rao S. S. Sadeghi P. Beaty J. Kavlock R. Ackerson K. (2001). Ambulatory 24-h colonic manometry in healthy humans. Am. J. Physiol. Gastrointest. Liver Physiol. 280, G629–G639. 11254489 10.1152/ajpgi.2001.280.4.G629

[B39] Rumessen J. J. Peters S. Thuneberg L. (1993). Light- and electron microscopical studies of interstitial cells of Cajal and muscle cells at the submucosal border of human colon. Lab. Invest. 68, 481–495. 8479156

[B40] Spencer N. J. Kyloh M. Wattchow D. A. Thomas A. Sia T. C. Brookes S. J.. (2012). Characterization of motor patterns in isolated human colon: are there differences in patients with slow-transit constipation? Am. J. Physiol. Gastrointest. Liver Physiol. 302, G34–G43. 10.1152/ajpgi.00319.2011 21960519

[B41] Taylor I. Duthie H. L. Smallwood R. Linkens D. (1975). Large bowel myoelectrical activity in man. Gut 16, 808–814. 10.1136/gut.16.10.808 1205275 PMC1413095

